# Dietary incorporation of brown seaweed spent oyster mushroom substrate alters growth performance, physiological responses and meat quality parameters in Boschveld roosters

**DOI:** 10.1038/s41598-024-65338-0

**Published:** 2024-06-22

**Authors:** Godfrey Mhlongo, Caven M. Mnisi

**Affiliations:** 1 Department of Animal Science, School of Agricultural Science, North-West University, Private Bag x2046, Mafikeng, 2745 South Africa; 2 Food Security and Safety Focus Area, Faculty of Natural and Agricultural Science, North-West University, Mafikeng, South Africa; 3 Faculty of Agriculture and Natural Sciences, School of Agricultural Sciences, University of Mpumalanga, Mbombela, South Africa

**Keywords:** Blood parameters, Chickens, Meat quality, Performance, Seaweed, White-rot fungi, Animal behaviour, Animal physiology

## Abstract

Use of brown seaweed (*Ecklonia maxima*) as a nutraceutical source in indigenous chicken diets is limited by high dietary fibre levels. Inoculating seaweeds with oyster mushroom (*Pleurotus ostreatus*) spawn (OMS) could enhance the utility of the spent mushroom substrate (SMS). This study investigated the effect of feeding incremental levels of brown seaweed SMS on growth performance, physiological responses, and meat quality parameters in Boschveld roosters. A total of 324, 4-week-old Boschveld roosters were weighed and randomly allotted to 36 pens (9 birds per pen) to produce six replicates per dietary treatment. The diets were formulated as follows: a standard grower diet (CON); and CON containing 150 g/kg of brown seaweed inoculated with OMS at 0 (SMS0), 20 (SMS20), 30 (SMS30), 40 (SMS40) and 50% (SMS50). Birds fed diet CON had the least feed intake (*p* < 0.05) than all the other SMS treatment levels in weeks 7, 8, 12, 14 and 15. Diet SMS40 promoted higher (*p* < 0.05) body weight gain (BWG) than CON in weeks 6, 7, 9 and 14. Gain-to-feed ratio linearly increased in weeks 7 [R^2^ = 0.288; *p* = 0.010], 11 [R^2^ = 0.581, *p* = 0.0001] and 14 [R^2^ = 0.389, *p* = 0.004], respectively. Quadratic responses (*p* < 0.05) were observed for BWG in week 5, white blood cells, heterophils, platelets, lymphocytes, monocytes, and relative spleen and large intestine weights as OMS levels increased. Linear increases were recorded for slaughter [R^2^ = 0.197, *p* = 0.017] and breast weights [R^2^ = 0.197, *p* = 0.020] as OMS levels increased. Diet SMS0 promoted higher (*p* < 0.05) relative caeca weights than the CON and SMS treatment groups. Neither quadratic nor linear responses (*p* > 0.05) were observed for breast meat quality parameters. In conclusion, feeding brown seaweed SMS improved growth performance and slaughter weight, altered some blood parameters and internal organs, without affecting breast meat quality of Boschveld roosters. Based on the quadratic response for BWG, the optimum OMS level was deduced at 20% in a brown seaweed-based Boschveld rooster diet.

## Introduction

The Boschveld chicken is a light red-brown hybrid cross of three indigenous chickens (Ovambo, Matabele, and Venda) that are used as a reliable protein source in rural South Africa^1^. This breed is characterized by firm meat texture, high fertility, disease resistance, thermo-tolerance, and strong eggshells^2^. However, their sustainable intensification is constrained by high feeding costs, poor feed conversion ratios, low growth rates, and later attainment of market weight and sexual maturity^3^. To combat high feeding costs, current research has focused on the utilization of locally available feed resources such as seaweeds^4^. Unlike conventional grains, nutraceutical sources such as brown seaweed (*Ecklonia maxima*) have no direct food value for humans and have been largely harvested to produce animal feed, fertilizers, plant bio-stimulants and alginate over the last few years^5^. Thus, their use in Boschveld chicken diets can be a strategy to deliver sustainable chicken production systems in South Africa^6^.

Brown seaweeds are rich sources of bioactive substances such as polyphenols, polysaccharides (alginate, agar, and carrageenan) and polyunsaturated fatty acids, which possess various biological functionalities with antiviral, antibacterial, antifungal, prebiotic, immunomodulatory, anti-inflammatory, and cholesterol-lowering effects^7,8^. These biomolecules have the potential to stimulate growth performance, promote immunological responses, and safeguard the chickens against debilitating pathogenic infections^6,9^.

However, the presence of lignin and non-starch polysaccharides (NSP) such as cellulose and hemicellulose^10^ could limit its utilization at higher dietary levels. Lignin and NSP hinder the chicken’s ability to digest feed, which affects the bioavailability of beneficial bioactive components resulting in poor growth performance^11^. Thus, lignin and NSP degradation strategies such as the use of oyster (*Pleurotus ostreatus*) mushroom mycelium or white-rot fungus can be used to reduce brown seaweed’s dietary fibre levels and enhance the utility of the spent mushroom substrate (SMS) in indigenous chicken diets.

Solid-state fermentation with fungi is widely recognized as an environmentally friendly and economically feasible technique for the bioconversion of lignin and NSP substrates^12^. White-rot fungi generate a group of ligninolytic enzymes such as lignin peroxidases, versatile peroxidases, manganese-dependent peroxidases and laccases^13^. These enzymes catalyse the oxidation of a variety of aromatic substrates, generating aromatic radicals, and altering the structure of raw materials containing lignocellulose^13^. Indeed, Mhlongo et al.^14^ reported that inoculating red grape pomace with varying levels (0, 20, 30, 40 and 50%) of oyster mushroom spawn improved crude protein (CP) while reducing the fibre content of the spent substrate. Thus, the inoculation of seaweeds with oyster mushroom spawn (OMS) could potentially facilitate the breakdown of lignocellulolytic matrix in seaweed, thereby enhancing the feed value of the SMS. Seaweed valorisation using OMS could improve the performance of Boschveld chickens while mitigating environmental challenges that are associated with the accumulation of seaweed heaps along offshore areas^15^. This study, therefore, investigated the effect of including incremental levels of brown seaweed SMS on growth performance, physiological and meat quality indices of Boschveld roosters. The study tested the hypothesis that the inoculation of brown seaweed with different levels of OMS will improve the utilisation of the SMS in diets of Boschveld roosters.

## Methods

### Ethical approval

The North-West University’s Animal Research Ethics Committee (approval number: NWU-00817-21-A5) approved the rearing and slaughter procedure of the roosters according to relevant established guidelines and regulations.

### Ingredients, spawn preparation, substrate inoculation and analysis

The brown seaweed (*Ecklonia maxima*) was purchased from RawKelp (PTY) LTD (Western Cape, South Africa), while the oyster mushroom (*Pleurotus ostreatus*) spawn was bought from Eco-Agro Enterprise (PTY) LTD (Nelspruit, South Africa). According to the company, a mature oyster mushroom is split in half and a small piece of mycelial tissue is extracted from the stem's top part. This tissue is placed aseptically on sterile potato dextrose agar (PDA) and incubated at 25 °C for 7–10 days to allow mycelium development. Sorghum grains (~ 100 g) are then soaked in a flask filled with water for overnight to prepare the spawn. The grains are then cooked/steamed for 45 min in an autoclave at 105 °C after which excess water was drained before cooling at room temperature. Thereafter, the grains are spread evenly on a polyethylene plastic to attain a moisture content of 50–54% and subsequently mixed with calcium carbonate as a nutrient supplement. The grain mixture is then packed in plastic bags and sterilized in an autoclave at 121 °C for 1 h to eliminate contaminants. The mycelial culture is then sub-cultured into the sterilized plastic bags inside a laminar airflow hood. The inoculated plastic bags are placed in an incubator at 24 ± 3 °C for 7 days, allowing the mycelium to colonize the grain substrate. The other feed ingredients were purchased from SimpleGrow Agric Services (PTY) LTD (Gauteng, South Africa). The brown seaweed samples were weighed (52 kg per treatment) into five containers (measuring 7000 cm^3^) and moistened with distilled water without allowing any water runoff^16^. Following the procedure outlined by Tuyen et al.^17^, the samples were subsequently sterilized in an autoclave (121 °C for 1 h at 100 kPa) and cooled at room temperature. Thereafter, the samples were inoculated with OMS at 0, 20, 30, 40 and 50%. The containers were then covered with black plastic bags and stored in a dark room to conserve moisture (a prerequisite for the reproduction of mushroom spores). The containers were stored at room temperature and relative humidity (60–70%) was recorded on a 2-day interval from day 1–35 of inoculation using a Thermo-Hygrometer (IN–OUT, Alla France Automatic Control Equipment Co., Ltd, PRC, Paris). To keep the surface moist and humid, all of the substrates in the containers were occasionally moistened^18^. At day 35 post inoculation, the remaining mycelial development was manually removed by hand from the SMS and air-dried at room temperature (27 °C) until constant weight was achieved. The dried SMS was milled (2 mm sieve; Polymix PX-MFC 90 D, Laufenburg, Switzerland) for laboratory analysis and diet formulation. The chemical composition (Table 1) of brown seaweed and SMS was analysed for dry matter (DM), organic matter, ash, ether extract (EE) and CP following methods described by AOAC^19^. The neutral detergent fibre (NDF) and acid detergent fibre (ADF) were analysed using the ANKOM^DELTA^ Automated Fibre Analyzer (ANKOM Technologies, NY, USA) following the detergent methods by van Soest et al.^20^. The ADF residue bags were then soaked in 72% H_2_SO_4_ for 3 h before being oven-dried at 105 °C for overnight to determine the acid detergent lignin (ADL). Metabolisable energy was calculated using the formula [ME (kcal/kg) = 2778–66 (Crude Fibre × 88% DM)] adapted from Masenya et al.^21^.

**Table 1:** Chemical composition and metabolizable energy values of brown seaweed substrate (g/kg DM, unless stated otherwise) without or with oyster mushroom spawn treatment.

Components	Substrates				
	S0	S20	S30	S40	S50
Dry matter (g/kg)	858.2	878.2	884.9	872.6	871.5
Ash	227.8	203.9	200.5	231.5	214.2
Organic matter	630.5	674.3	684.3	659.2	657.3
ME (Kcal/Kg)	2754.0	2757.0	2755.0	2758.0	2757.0
Crude fibre	421.6	359.9	396.5	346.7	366.8
Neutral detergent fibre	340.7	322.1	321.3	307.9	304.0
Acid detergent fibre	288.3	247.0	246.91	237.2	234.0
Acid detergent lignin	140.7	114.5	114.3	104.4	80.65
Crude protein	111.7	115.7	115.0	115.5	111.8
Ether extract	285.1	279.3	263.8	286.0	261.7
Calcium	27.89	26.14	28.80	32.27	29.42
Potassium	23.73	20.86	20.31	21.99	22.09
Magnesium	9.24	7.78	7.78	8.44	8.43
Sodium	27.45	23.71	23.32	24.64	25.07
Phosphorus	1.59	1.68	1.62	1.75	1.68
Arginine (g/100 g DM)	0.28	0.34	0.38	0.39	0.35
Methionine (g/100 g DM)	0.21	0.21	0.21	0.26	0.22
Threonine (g/100 g DM)	0.60	0.57	0.60	0.59	0.58
Lysine (g/100 g DM)	0.70	0.61	0.66	0.60	0.73
Leucine (g/100 g DM)	0.54	0.53	0.55	0.51	0.55

^1^Substrates: S0 = brown seaweed without oyster mushroom spawn, S20 = brown seaweed inoculated with 20% oyster mushroom spawn, S30 = brown seaweed inoculated with 30% oyster mushroom spawn, S40 = brown seaweed inoculated with 40% oyster mushroom spawn, and S50 = brown seaweed inoculated with 50% oyster mushroom spawn.

### Diet formulation and analysis

Six experimental diets were formulated (Table 2) to meet the nutritional requirements of Boschveld chickens^22^ using Spesfeed Express nutritional software. The diets were as follows: CON = a standard grower diet; SMS0 = CON containing 150 g/kg brown seaweed without OMS; SMS20 = CON containing 150 g/kg of brown seaweed substrate after being inoculated with 20% OMS; SMS30 = CON containing 150 g/kg of brown seaweed substrate after being inoculated with 30% OMS; SMS40 = CON containing 150 g/kg of brown seaweed substrate after being inoculated with 40% OMS; and SMS50 = CON containing 150 g/kg of brown seaweed substrate after being inoculated with 50% OMS. The diets were in a mash form and were formulated by hand mixing. The ingredient composition of the diets was analysed as described above for the SMS.

**Table 2:** Ingredient composition (g/kg *as fed basis*, unless stated otherwise) of experimental diets.

Ingredients	^1^Experimental diets					
	CON	SMS0	SM20	SM30	SM40	SM50
SMS	0	150.0	150.0	150.0	150.0	150.0
Yellow maize	645.05	476.89	476.89	476.89	476.89	476.89
Soya oil cake (46%)	311.74	314.07	314.07	314.07	314.07	314.07
Soya crude oil	10.0	40.14	40.14	40.14	40.14	40.14
Limestone powder	14.09	0	0	0	0	0
Monocalcium phosphate	7.01	7.64	7.64	7.64	7.64	7.64
Salt (Fine)	3.45	3.53	3.53	3.53	3.53	3.53
DL-Methionine	3.20	3.32	3.32	3.32	3.32	3.32
L-Threonine	0.62	0.49	0.49	0.49	0.49	0.49
Lysine HCL	2.23	1.33	1.33	1.33	1.33	1.33
^2^Premix	2.50	2.50	2.50	2.50	2.50	2.50
Axtraphytase (10,000 FTU/g)	0.10	0.10	0.10	0.10	0.10	0.10
Nutritional composition (g/kg DM, unless stated otherwise)						
Dry matter (g/kg)	894.7	899.5	903.2	898.9	923.2	924.2
Calculated ME (Kcal/kg)	2776.0	2774.0	2774.0	2774.0	2774.0	2774.0
Crude fibre	33.14	66.17	63.42	76.57	65.40	67.26
Ash	54.13	66.99	67.59	61.63	56.51	73.80
Organic matter	840.6	832.5	835.7	837.3	866.7	866.7
NDF	109.3	145.3	144.6	136.7	129.4	124.7
ADF	44.80	67.59	68.12	66.26	64.39	62.10
ADL	4.78	9.99	8.35	7.45	6.34	5.05
Crude protein	205.1	219.6	220.2	220.1	231.3	220.4
Ether extract	271.0	459.7	380.0	311.3	345.2	264.2
Calcium	7.37	6.27	7.63	8.07	6.94	6.52
Potassium	8.29	12.94	13.61	12.50	12.12	12.78
Magnesium	1.48	2.55	2.67	2.66	2.54	2.60
Sodium	1.38	5.34	5.66	5.33	4.83	5.37
Phosphorus	4.98	4.72	5.36	5.12	4.54	4.73
Arginine (g/100 g)	1.68	1.72	1.85	1.73	1.59	1.66
Methionine (g/100 g)	0.75	0.94	0.69	0.84	0.63	0.85
Threonine (g/100 g)	0.97	1.04	1.01	1.07	0.93	0.98
Lysine (g/100 g)	1.23	1.71	1.13	1.16	1.15	1.41
Leucine (g/100 g)	1.75	1.94	1.91	1.95	1.66	1.76

^1^Experimental diets: CON = a standard grower diet, SMS0 = CON containing 150 g/kg brown seaweed without oyster mushroom spawn inoculation, SMS20 = CON containing 150 g/kg of brown seaweed substrate after inoculating with 20% oyster mushroom spawn, SMS30 = CON containing 150 g/kg of brown seaweed substrate after inoculating with 30% oyster mushroom spawn, SMS40 = CON containing 150 g/kg of brown seaweed substrate after inoculating with 40% oyster mushroom spawn, and SMS50 = CON containing 150 g/kg of brown seaweed substrate after inoculating with 50% oyster mushroom spawn. ^2^Premix: 79 mg zinc sulphate; 8.0 mg copper sulphate; 30 mg niacin; 80 mg ferrous sulphate; 11 000 IU vitamin A; 100 mg magnesium sulphate; 0.34 mg potassium iodine; 10 mg pantothenic acid; 0.7 mg folic acid; 5.1 mg vitamin B6; 0.12 g biotin; 2.5 mg vitamin B1; 2500 IU vitamin D3; 2.0 mg vitamin K3; 4.5 mg vitamin B2; 25 IU vitamin E; and 0.25 mg sodium selenite. ME = metabolizable energy; NDF = neutral detergent fibre; ADF = acid detergent fibre; ADL = acid detergent lignin.

### Feeding trial and management

A total of 800, 1-week-old Boschveld chicks were purchased from Boschveld Ranching (PTY) LTD (Bela-Bela, Limpopo, South Africa). For the first 3 days, the birds were offered a stress pack (Phenix stresspac, Virbac, South Africa) and were subsequently reared on a commercial starter diet for 3 weeks in North-West University Research farm (Molelwane; (33° 54′ 55″ S, 18° 23′ 33″ E). The birds were sexed in Molelwane farm and only the males (n = 324) were used for the feeding trial, which was carried out in summer (November 2022−January 2023) at Rooigrond Commercial Farm (25° 55′ 0″ S; 25° 48′ 0″ E; North West, South Africa). The roosters were accustomed to the experimental diets for 3 days before the commencement of measurements at 4 wk of age. In a completely randomized design, the 4 wk-old roosters were randomly allocated to 36 floor pens (experimental units in the same chicken house) such that each of the 6 experimental diets had 6 replicate groups. The pen (measuring 1.5 m Length × 1.5 m Width × 1.7 m Height) carried 9 birds and the floor was covered with sunflower husks. The birds were reared under natural lighting from morning (06h00) to evening (18h00). Fresh clean water and the diets were offered daily using Poltek water fountain (10L, NWK, Mafikeng, South Africa) and Poltek tube feeders (3/100, NWK, Mafikeng, South Africa) without any restrictions.

### Growth performance

Initial live weights (310.8 ± 13.98 g) of all the birds per pen were recorded and then weighed weekly (from week 4 to week 15) to calculate average weekly body weight gain. Feed intake was calculated based on the amount of feed offered and feed refusal. The gain-to-feed ratio (GF) was calculated as a proportion of weight gain and feed consumption. During the feeding trial, mortality was recorded and was used to correct the growth performance data.

### Blood collection and analysis

At 14 weeks and 5 days of age, fresh blood samples (4 mL) were randomly collected from two birds per pen via the brachial vein using sterilized 5 mL syringes and 23-gauge needles. The whole blood (with EDTA) tubes were kept in a cooler and were analysed within 48 h of collection^23^. Haematological parameters viz., haematocrits, monocytes, platelets, white blood cell (WBC), lymphocytes and heterophils were analysed using an automated LaserCyte Haematology Analyser (IDEXX Laboratories (Pty) Ltd., Gauteng, South Africa). Serum tubes without any anticoagulant were centrifuged (Cryste Varispin 4 Multipurpose centrifuge, Cryste CO. LTD, Bucheon, South Korea) after blood collection at 14,989 relative centrifugal force (RCF-g) for 5 min^23^. Serum biochemical parameters viz., calcium, total protein, symmetric dimethyl arginine (SDMA), albumin, glucose, creatinine, blood urea nitrogen (BUN), alanine aminotransferase (ALT), lipase, phosphorus, alkaline phosphatase (ALKP), cholesterol and amylase were analysed using an automated Catalyst One Chemistry Analyzer (IDEXX Laboratories (Pty) Ltd., Gauteng, South Africa).

### Slaughter and post-mortem measurements

At 15 weeks of age, all the roosters were weighed to determine their final slaughter weights. A total of 7 birds per pen were stunned and slaughtered by exsanguination at a commercial abattoir in the study site. Immediately after dressing and evisceration, the carcasses were weighed using a digital scale (Model 330 Weighing, Richter Scale (Pty) Ltd., Gauteng, South Africa) to determine hot carcass weight (HCW) and then reweighed after chilling at 16 °C for 24 h to determine cold carcass weight (CCW). The weights of the drumstick, breast, wing and thigh were measured using the weighing scale stated above. The dressing percentage was calculated as the proportion of HCW to slaughter weight. The weights of the cleaned gizzard, spleen, proventriculus, liver, small intestines, duodenum, jejunum, ileum, large intestines caecum and colon were determined using an analytical scale (Explorer EX224, OHAUS Corp, NJ).

Breast meat pH was measured at 1 h and 24 h post-slaughter using a digital meter equipped with a spear-piercing electrode (HI98163 Professional Portable pH Meter, Hanna Instruments (Pty) Ltd, Johannesburg, South Africa), which was calibrated using standard solutions (pH 4, 7 and 10) after measuring each replicate pen. Breast meat lightness (L*), redness (a*) and yellowness (b*) were determined using a spectrophotometer (Konica Minolta Chroma Meter CR-400, Narich (Pty) Ltd, Tokyo, Japan) at 1 and 24 h post-mortem. The breast meat water-holding capacity (WHC) was determined using the filter-paper method by Whiting and Jenkins^24^. The method developed by Honikel^25^ was used to measure drip loss, where breast meat samples were hooked and suspended from a wire steel for 72 h at 4 °C. Cooking losses were determined by cooking pre-weighed breast meat samples to a core temperature of 75°C^25^. To determine the shear force, raw breast meat samples were sheared using a Meullenet-Owens Razor Shear Blade (A/MORS) installed in a Texture Analyzer (TA.XT plus, Stable Micro Systems, Surrey, UK).

### Statistical analysis

The growth performance, physiological and meat quality data was examined for normality using the normal option in the PROC Univariate statement in SAS version 9.4^26^. Repeated measures analysis in PROC GLM of SAS^26^ was used to determine the interaction effect between time and diet on average FI, BWG, and GF data. One-way analysis of variance was used to account for dietary differences in performance, physiological and meat quality data (PROC GLM; SAS^26^). The probability of difference option in SAS was used to separate the treatment means. Polynomial contrasts were used to evaluate data for linear and quadratic coefficients in response to incremental levels of OMS by means of PROC RSREG in SAS. Pre-planned orthogonal contrast statements were used to compare the performance of CON against SMS0. The CON data was not used for the regression analysis. For all the statistical tests, significance was considered at *p* < 0.05.

### Animal welfare statement

The authors certify that they have complied with the journal's ethical policies, as stated on the author guidelines page, and that they have obtained the necessary approval from the ethical review committee. The authors affirm that they complied with EU regulations on the care of animals used for research purposes.

### Research involving animals

The study was conducted following ARRIVE guidelines.

## Results

### Composition of the substrates and diets

Table 1 demonstrates that inoculating brown seaweed with the OMS tended to enhance the DM, calcium, phosphorus and arginine content as the SMS levels increased. However, the incremental levels of SMS tended to reduce the crude fibre, NDF, ADF and ADL contents of SMS. Table 2 shows that incorporating SMS in the experimental diets tended to increase the DM, calcium and arginine content as SMS levels increased. Higher inclusion of the SMS tended to reduce the NDF, ADF and ADL content of the diets. The ME and CP remained the same in both SMS and the experimental diets.

### Growth performance

There were no dietary effects on mortality (*p* = 0.183) with the means ranging from 1.698 to 10.19%. Repeated measures analysis revealed significant week × diet interaction effects on FI (*p* = 0.0001), BWG (*p* = 0.0001) and GF (*p* = 0.0001). Table 3 indicates that there were neither linear nor quadratic effects (*p* > 0.05) for the FI, except for week 13 [*y* = 716.9 (± 26.50)–1.653 (± 2.293) *x*; R^2^ = 0.212, *p* = 0.012] where a linear decrease was observed for FI in response to increasing OMS levels. In weeks 7, 8, 11, 12, 14 and 15, the CON diet promoted the least (*p* < 0.05) FI than the other treatment groups, which were similar (*p* > 0.05). In week 9, diet SMS40 promoted the highest FI (*p* < 0.05), followed by SMS0, SMS20, SMS30 and SMS50, and the lowest FI was from those reared on CON. In week 11, the CON diet promoted the lowest FI (578.5 g/bird) than the SMS0 and SMS50 diets but were all similar (*p* > 0.05) to those fed the SMS20, SMS30 and SMS40 diets. Orthogonal contrasts showed that SMS0 promoted a higher (*p* < 0.05) FI in weeks 6–15 than CON, with the exception of week 13.

**Table 3:** Average weekly feed intake (g/bird) of Boschveld roosters fed with diets containing brown seaweed spent oyster mushroom substrate.

	^1^Experimental diets						^2^SEM	Significance		
	CON	SMS0	SMS20	SMS30	SMS40	SMS50		*P*-value	Linear	Quadratic
Week 5	164.6	192.3	189.6	187.7	172.2	190.0	11.21	0.411	0.513	0.691
Week 6	319.8	372.3	360.6	369.1	359.9	360.4	9.034	0.051	0.366	0.841
Week 7	347.2^a^	417.4^b^	409.1^b^	428.5^b^	417.7^b^	417.5^b^	10.42	0.0001	0.812	0.911
Week 8	387.2^a^	486.0^b^	467.3^b^	493.0^b^	501.0^b^	495.9^b^	11.14	0.0001	0.222	0.429
Week 9	436.3^a^	535.2^b^	517.8^b^	530.0^b^	544.2^c^	530.8^b^	13.65	0.0001	0.798	0.621
Week 10	509.4	561.7	539.4	535.0	548.4	557.3	15.18	0.206	0.828	0.167
Week 11	578.5^a^	646.9^b^	634.4^ab^	628.2^ab^	624.8^ab^	646.2^b^	15.41	0.041	0.723	0.302
Week 12	523.1^a^	602.9^b^	614.4^b^	580.3^ab^	607.6^b^	608.6^b^	17.83	0.009	0.952	0.690
Week 13	667.5	717.0	683.0	658.5	648.6	620.7	26.74	0.224	0.012	0.917
Week 14	596.0^a^	755.3^b^	753.0^b^	850.1^b^	840.3^b^	774.3^b^	27.87	0.0001	0.187	0.198
Week 15	557.9^a^	707.2^b^	662.0^b^	726.4^b^	711.5^b^	718.8^b^	17.03	0.0001	0.348	0.344

^a,b^ Means, in a row, with different superscripts denote statistical differences (*p* < 0.05).^1^Experimental diets: CON = a standard grower diet, SMS0 = CON containing 150 g/kg brown seaweed without oyster mushroom spawn inoculation, SMS20 = CON containing 150 g/kg of brown seaweed substrate after inoculating with 20% oyster mushroom spawn, SMS30 = CON containing 150 g/kg of brown seaweed substrate after inoculating with 30% oyster mushroom spawn, SMS40 = CON containing 150 g/kg of brown seaweed substrate after inoculating with 40% oyster mushroom spawn, and SMS50 = CON containing 150 g/kg of brown seaweed substrate after inoculating with 50% oyster mushroom spawn. ^2^SEM = standard error of the mean.

Table 4 indicates that there were linear increases (*p* > 0.05) for BWG in week 8 [*y* = 0.396 (± 0.549) *x* + 143.9 (± 5.192); R^2^ = 0.396, *p* = 0.012], week 11 [*y* = 0.769 (± 0.806) *x* + 128.9 (± 7.621); R^**2**^ = 0.531, *p* = 0.002], week 14 [*y* = 3.819 (± 2.192)* x* + 77.25 (± 20.74); R^2^ = 0.407, *p* = 0.014], and week 15 [*y* = 2.525 (± 1.732) *x* + 133.4 (± 16.38); R^2^ = 0.473, *p* = 0.005]. A negative linear effect was observed for BWG in week 13 [*y* = 164.9 (± 14.60)–0.850 (± 1.543) *x*; R^2^ = 0.137, *p* = 0.049] as dietary OMS levels increased. There was a positive quadratic effect for BWG in week 5 [*y* = 0.050 (± 0.018) *x*^2^–2.119 (± 0.788)* x* + 83.98 (± 7.454); R^2^ = 0.377, *p* = 0.015; optimised weight gain at 20% SMS]. In weeks 6 and 7, birds fed CON promoted lower BWG (132.8 g/bird) than diet SMS40 (150.2 g/bird), but were all similar (*p* > 0.05) to those fed the SMS0, SMS20, SMS30 and SMS50. In week 9, diet SMS40 (180.0 g/bird) promoted the highest BWG than diets CON and SMS20, which were similar (*p* > 0.05) to the SMS0, SMS30, SMS40 and SMS50 diets. In week 10, diet CON (168.8 g/bird) promoted the highest BWG than all the other treatment groups. In week 11, birds fed the SMS30, SMS40 and SMS50 had higher (*p* < 0.05) BWG than those fed the CON, SMS0 and SMS20 diets. In week 14, the CON diet promoted the lowest BWG (55.88 g/bird) than diets SMS30 and SMS40. In week 15, CON promoted the lowest (117.3 g/bird) BWG than diets SMS30, SMS40 and SMS50, which were all similar (*p* > 0.05) to diets SMS0 and SMS20. Relative to CON, SMS0 promoted higher (*p* < 0.05) BWG in weeks 5, 9, 10 and 14.

**Table 4:** Average weekly body weight gain (g/bird) of Boschveld roosters fed with diets containing brown seaweed spent oyster mushroom substrate.

	^1^Experimental diets						^2^SEM	Significance		
	CON	SMS0	SMS20	SMS30	SMS40	SMS50		*P*-value	Linear	Quadratic
Week 5	54.49	81.31	70.42	68.24	65.46	94.70	8.404	0.054	0.906	0.015
Week 6	132.8^a^	133.8^ab^	141.9^ab^	147.8^ab^	150.2^b^	135.6^ab^	4.077	0.011	0.125	0.176
Week 7	143.6^a^	147.1^ab^	147.2^ab^	156.7^ab^	161.0^b^	151.3^ab^	4.060	0.044	0.234	0.294
Week 8	153.4	150.1	148.5	160.0	171.9	159.2	4.850	0.084	0.012	0.822
Week 9	162.1^ab^	173.9^bc^	155.7^ab^	178.3^bc^	180.0^c^	174.3^bc^	4.256	0.004	0.919	0.556
Week 10	168.8^b^	143.4^a^	136.1^a^	130.3^a^	134.9^a^	140.2^a^	5.814	0.001	0.082	0.776
Week 11	139.4^a^	133.4^a^	137.0^a^	166.9^b^	178.3^b^	179.1^b^	6.071	0.0001	0.002	0.773
Week 12	110.2	91.22	91.50	83.75	85.21	80.77	10.02	0.475	0.545	0.377
Week 13	159.3	167.5	145.6	154.5	163.7	115.1	15.59	0.217	0.024	0.776
Week 14	55.88^a^	86.12^ab^	114.2^abc^	165.1^c^	168.3^c^	135.0^bc^	14.95	0.0001	0.014	0.402
Week 15	117.3^a^	140.5^ab^	167.3^ab^	190.6^b^	184.9^b^	206.2^b^	16.65	0.007	0.005	0.707

^a–c^Means, in a row, with different superscripts denote statistical differences (*p* < 0.05). ^1^Experimental diets: CON = a standard grower diet, SMS0 = CON containing 150 g/kg brown seaweed without oyster mushroom spawn inoculation, SMS20 = CON containing 150 g/kg of brown seaweed substrate after inoculating with 20% oyster mushroom spawn, SMS30 = CON containing 150 g/kg of brown seaweed substrate after inoculating with 30% oyster mushroom spawn, SMS40 = CON containing 150 g/kg of brown seaweed substrate after inoculating with 40% oyster mushroom spawn, and SMS50 = CON containing 150 g/kg of brown seaweed substrate after inoculating with 50% oyster mushroom spawn.

^2^SEM = Standard error of the mean.

Table 5 indicates that there were positive and negative quadratic effects (*p* < 0.05) observed for GF in week 5 [*y* = 0.0002 (± 0.0001) *x*^2^–0.007 (± 0.003) *x* + 0.430 (± 0.029); R^2^ = 0.320; *p* = 0.006] and week 6 [*y* = 0.346 (± 0.012)–0.004 (± 0.001) *x*–0.0001 (± 0.00002) *x*^2^; R^2^ = 0.383, *p* = 0.025], respectively. There were linear increases (*p* < 0.05) for GF in week 7 [*y* = 0.001 (± 0.001) *x* + 0.351 (± 0.006); R^2^ = 0.288, *p* = 0.010], week 11 [*y* = 0.002 (± 0.001) *x* + 0.201 (± 0.011); R^2^ = 0.581, *p* = 0.0001] and week 14 [*y* = 0.004 (± 0.002) *x* + 0.109 (± 0.017); R^2^ = 0.389, *p* = 0.004]. However, a linear decline was recorded for GF in week 12 [*y* = 0.150 (± 0.022)–0.0001 (± 0.002) *x*; R^2^ = 0.271, *p* = 0.015]. In week 5, diet SMS50 promoted the highest GF than CON, SMS20 and SMS30 diets, which were all similar (*p* > 0.05) to diets SMS0 and SMS40. In week 6, diet SMS40 promoted the highest GF (0.418) while diet SMS0 resulted in the lowest GF value (0.347). However, all the treatment groups were similar (*p* > 0.05) to the CON group. In week 7, the CON and SMS40 diets promoted the highest GF than those fed the SMS0, SMS20 and SMS50 diets, which were all similar (*p* > 0.05) to diet SMS30. In weeks 8 and 10, the CON diet promoted the highest GF than all the other treatment groups. In week 9, the CON and SMS40 diets promoted the highest GF than diets SMS0, SMS20 and SMS50 diets. In week 11, diets SMS40 and SMS50 promoted the highest (*p* < 0.05) GF than the SMS0 and SMS20 diets, which were similar (*p* > 0.05) to the CON. In week 12, CON promoted the highest GF than diets SMS30, SMS40 and SMS50 but had similar (*p* > 0.05) GF as birds fed diets SMS0 and SMS20. In week 15, the CON diet promoted the least GF than diets SMS30 and SMS40. Relative to CON, SMS0 promoted higher (*p* < 0.05) GF in weeks 6–12.

**Table 5:** Average weekly gain-to-feed ratio of Boschveld roosters fed with diets containing brown seaweed spent oyster mushroom substrate.

	^1^Experimental diets						^2^SEM	Significance		
	CON	SMS0	SMS20	SMS30	SMS40	SMS50		*P*-value	Linear	Quadratic
Week 5	0.353^a^	0.426^ab^	0.370^a^	0.367^a^	0.397^ab^	0.503^b^	0.032	0.042	0.421	0.006
Week 6	0.400^abc^	0.347^a^	0.395^abc^	0.401^bc^	0.418^c^	0.364^ab^	0.013	0.006	0.014	0.025
Week 7	0.388^b^	0.352^a^	0.361^a^	0.366^ab^	0.385^b^	0.362^a^	0.005	0.0004	0.010	0.499
Week 8	0.387^b^	0.308^a^	0.319^a^	0.323^a^	0.343^a^	0.321^a^	0.009	0.0001	0.110	0.501
Week 9	0.372^b^	0.326^a^	0.318^a^	0.344^b^	0.331^a^	0.328^a^	0.008	0.0009	0.466	0.974
Week 10	0.332^b^	0.255^a^	0.253^a^	0.244^a^	0.245^a^	0.245^a^	0.009	0.0001	0.315	0.578
Week 11	0.241^ab^	0.206^a^	0.217^a^	0.266^bc^	0.286^c^	0.278^bc^	0.011	0.0001	0.0001	0.880
Week 12	0.211^b^	0.152^ab^	0.127^ab^	0.103^a^	0.09^a^	0.088^a^	0.023	0.007	0.015	0.404
Week 13	0.239	0.233	0.212	0.230	0.234	0.232	0.020	0.865	0.500	0.068
Week 14	0.094^a^	0.114^ab^	0.150^abc^	0.193^c^	0.199^c^	0.171^bc^	0.015	0.0001	0.004	0.118
Week 15	0.210	0.198	0.254	0.262	0.261	0.288	0.024	0.099	0.052	0.064

^a–c^ Means, in a row, with different superscripts denote statistical differences (*p* < 0.05). ^1^Experimental diets: CON = a standard grower diet, SMS0 = CON containing 150 g/kg brown seaweed without oyster mushroom spawn inoculation, SMS20 = CON containing 150 g/kg of brown seaweed substrate after inoculating with 20% oyster mushroom spawn, SMS30 = CON containing 150 g/kg of brown seaweed substrate after inoculating with 30% oyster mushroom spawn, SMS40 = CON containing 150 g/kg of brown seaweed substrate after inoculating with 40% oyster mushroom spawn, and SMS50 = CON containing 150 g/kg of brown seaweed substrate after inoculating with 50% oyster mushroom spawn. ^2^SEM = standard error of the mean.

### Haematological and serum biochemical parameters

There were positive quadratic effects (*p* < 0.05) observed for white blood cell (WBC) count and lymphocytes. However, negative quadratic effects were observed for heterophils, platelets and monocytes in response to the increasing dietary OMS levels (Table S1).

Table 6 indicates that there were neither linear nor quadratic effects for serum biochemical parameters, except for albumin which linearly improved [*y* = 0.010 (± 0.005) *x* + 5.816 (± 0.056); R^2^ = 0.259, *p* = 0.043] with dietary OMS levels. Dietary treatments influenced (*p* < 0.05) WBC, heterophils, platelets, lymphocytes, and monocytes. Birds fed with diet SMS50 had higher (*p* < 0.05) WBC than those fed with diets SMS0 and SMS20. Birds fed with CON had similar (*p* > 0.05) WBC levels as those fed with diets SMS30, SMS40 and SMS50. SMS30 promoted higher (*p* < 0.05) heterophils than CON but were similar to the other treatment groups. The CON and SMS40 diets resulted in the least platelets compared to all the other dietary treatment groups. Birds reared on CON and SMS50 diets had higher (*p* < 0.05) lymphocytes than those reared on the other treatment groups. Diet SMS20 promoted higher (*p* < 0.05) monocytes than diet SMS50, which were similar (*p* > 0.05) to the other treatment groups. Relative to CON, SMS0 resulted in lower WBC (*p* = 0.014), lymphocytes (*p* = 0.0001), blood urea nitrogen (*p* = 0.038) and amylase (*p* = 0.031) concentrations. However, SMS0 produced higher platelets (*p* = 0.003) and heterophils (*p* = 0.0001) concentrations than the CON.

**Table 6:** Haematological and serum biochemical parameters of Boschveld roosters fed with diets containing brown seaweed spent oyster mushroom substrate.

^2^Parameters	^1^Experimental diets						^3^SEM	Significance		
	CON	SMS0	SMS20	SMS30	SMS40	SMS50		*P*-value	Linear	Quadratic
Haematocrit (%)	34.83	33.25	34.75	35.42	33.33	33.58	0.823	0.313	0.989	0.068
^2^WBC (× 10^9^/L)	15.23^bc^	11.50^a^	11.33^a^	12.92^ab^	12.33^ab^	17.45^c^	0.975	0.0002	0.0003	0.008
Platelets (× 10^9^/L)	21.83^a^	39.50^b^	38.67^b^	38.50^b^	37.92^b^	26.56^a^	2.222	0.0001	0.001	0.008
Heterophils (× 10^9^/L)	5.621^a^	7.416^ab^	7.987^ab^	8.271^b^	7.700^ab^	5.867^ab^	0.769	0.031	0.435	0.024
Lymphocytes (%)	54.17^b^	20.00^a^	17.08^a^	24.17^a^	18.25^a^	54.25^b^	3.879	0.0001	0.0003	0.0006
Monocytes (%)	9.75^ab^	11.00^ab^	13.83^b^	11.50^ab^	11.17^ab^	7.75^a^	1.194	0.032	0.054	0.007
Glucose (mmol/L)	131.8	122.1	127.3	116.8	127.5	138.6	5.279	0.099	0.642	0.501
SDMA (µg/dL)	28.00	21.50	25.42	23.42	25.58	27.17	3.219	0.733	0.347	0.899
Creatinine (µmol/dL)	0.100	0.100	0.100	0.100	0.133	0.100	0.014	0.435	0.297	0.848
BUN	4.75	3.54	4.33	3.92	4.33	4.42	0.446	0.492	0.379	0.635
Phosphorus (mmol/L)	16.10	16.10	16.10	15.59	15.45	15.76	0.321	0.544	0.297	0.848
Calcium (mmol/L)	5.08	5.15	5.30	5.29	5.21	5.31	0.153	0.868	0.377	0.328
Albumin (g/L)	6.0	5.81	6.0	5.68	5.86	6.0	0.132	0.439	0.043	0.228
Cholesterol (mmol/L)	33.58	34.75	29.17	24.58	33.00	27.92	7.556	0.926	0.501	0.637
Amylase (U/L)	566.1	455.8	504.5	487.0	484.8	513.6	37.01	0.431	0.984	0.123
ATL (U/L)	104.1	76.83	93.33	74.50	75.63	88.50	12.25	0.602	0.914	0.413
ALKP (U/L)	9.167	10.00	8.333	9.000	8.000	9.167	0.863	0.681	0.139	0.724
Lipase (U/L)	53.50	65.00	50.58	51.50	44.75	53.00	8.745	0.718	0.124	0.931

^a-c^ Means, in a row, with different superscripts denote statistical differences (*P* < 0.05). ^1^Experimental diets: CON = a standard grower diet, SMS0 = CON containing 150 g/kg brown seaweed without oyster mushroom spawn inoculation, SMS20 = CON containing 150 g/kg of brown seaweed substrate after inoculating with 20% oyster mushroom spawn, SMS30 = CON containing 150 g/kg of brown seaweed substrate after inoculating with 30% oyster mushroom spawn, SMS40 = CON containing 150 g/kg of brown seaweed substrate after inoculating with 40% oyster mushroom spawn, and SMS50 = CON containing 150 g/kg of brown seaweed substrate after inoculating with 50% oyster mushroom spawn.

^2^Parameters: WBC =  white blood cell, SDMA =  symmetric dimethylarginine, BUN =  blood urea nitrogen, ALT =  alanine aminotransferase, ALKP =   alkaline phosphatase. ^3^SEM = standard error of the mean.

### Carcass characteristics and internal organs

Table 7 shows that there were linear increases for slaughter weight [*y* = 2.76 (± 2.649) *x* + 1641.8 (± 30.61); R^2^ = 0.197, *p* = 0.017] and breast weight [*y* = 0.048 (± 0.034) *x* + 8.48 (± 0.395); R^2^ = 0.197, *p* = 0.020] as OMS levels increased. Caecum weight linearly declined [*y* = 1.048 (± 0.036)–0.005 (± 0.003) *x*; R^2^ = 0.176; *p* = 0.035] with increasing OMS levels. A positive quadratic effect was observed for spleen weight [*y* = 0.0001 (± 0.0001) *x*^2^–0.01 (± 0.003) *x* + 0.350 (± 0.033); R^2^ = 0.238, *p* = 0.023], whereas a negative quadratic effect was observed for the large intestine weight [*y* = 0.151 (± 0.014) + 0.003 (± 0.001) *x*–0.0001 (± 0.00002) *x*^2^; R^2^ = 0.210, *p* = 0.015]. There were significant dietary effects on slaughter weight, where CON, SMS0 and SMS20 diets promoted the least (*p* < 0.05) slaughter weight than diet SMS30, SMS40, and SMS50. Diet SMS0 promoted higher caeca weight (1.045% CCW) than CON (0.864% CCW) but was similar (*p* > 0.05) to SMS20, SMS40 and SMS50 diets. Relative to CON, SMS0 promoted a larger gizzard (*p* = 0.036) and caeca weights (*p* = 0.009) but the rest of the carcass cuts and internal organs were not different (*p* > 0.05).

**Table 7:** Carcass characteristics and internal organs (% CCW, unless stated otherwise) of Boschveld roosters fed with diets containing brown seaweed spent oyster mushroom substrate.

^2^Parameters	^1^Experimental diets						^3^SEM	Significance		
	CON	SMS0	SMS20	SMS30	SMS40	SMS50		*P*-value	Linear	Quadratic
Slaughter weight (g)	1640.8^a^	1653.0^a^	1646.4^a^	1734.9^b^	1762.7^b^	1722.2^ab^	28.69	0.015	0.017	0.784
HCW (g)	1208.0	1210.0	1197.1	1256.9	1202.9	1234.1	33.16	0.794	0.621	0.972
CCW (g)	1172.7	1173.0	1163.4	1214.5	1179.0	1195.1	31.22	0.873	0.556	0.927
Dressing (%)	73.60	73.23	72.64	72.42	69.96	71.70	1.562	0.630	0.259	0.909
Breast	9.939	8.470	9.193	9.778	9.381	9.856	0.442	0.197	0.020	0.498
Drumstick	7.383	7.635	7.413	7.639	7.600	7.449	0.140	0.626	0.630	0.983
Thigh	6.092	6.412	6.308	6.228	6.414	6.237	0.139	0.570	0.391	0.720
Wing	7.946	7.746	7.917	7.585	8.006	7.817	0.204	0.719	0.719	0.990
Liver	2.053	2.230	2.048	2.088	1.992	2.137	0.066	0.196	0.170	0.075
Gizzard	3.063	3.651	3.744	3.650	3.440	3.609	0.156	0.052	0.444	0.767
Spleen	0.271	0.352	0.231	0.241	0.240	0.282	0.033	0.135	0.117	0.023
Proventriculus	0.456	0.482	0.532	0.504	0.462	0.534	0.022	0.059	0.573	0.921
Duodenum	1.049	1.063	1.083	1.099	1.117	1.120	0.039	0.734	0.201	0.992
Jejunum	1.467	1.577	1.614	1.501	1.503	1.542	0.059	0.527	0.372	0.917
Ileum	1.178	1.256	1.298	1.250	1.359	1.309	0.060	0.397	0.325	0.993
Caecum	0.864^a^	1.045^c^	1.001^bc^	0.901^ab^	0.981^abc^	0.936^abc^	0.033	0.005	0.035	0.368
Small intestines	3.694	3.896	3.995	3.851	3.979	3.972	0.121	0.495	0.682	0.955
Large intestines	0.165	0.149	0.191	0.179	0.151	0.145	0.014	0.166	0.548	0.015

^a,b^ Means, in a row, with different superscripts denote statistical differences (*P* < 0.05). ^1^Experimental diets: CON = a standard grower diet, SMS0 = CON containing 150 g/kg brown seaweed without oyster mushroom spawn inoculation, SMS20 = CON containing 150 g/kg of brown seaweed substrate after inoculating with 20% oyster mushroom spawn, SMS30 = CON containing 150 g/kg of brown seaweed substrate after inoculating with 30% oyster mushroom spawn, SMS40 = CON containing 150 g/kg of brown seaweed substrate after inoculating with 40% oyster mushroom spawn, and SMS50 = CON containing 150 g/kg of brown seaweed substrate after inoculating with 50% oyster mushroom spawn. ^2^Parameters: HCW = hot carcass weight, CCW = cold carcass weight. ^3^SEM = standard error of the mean.

### Meat quality parameters

Table 8 shows that there were neither linear nor quadratic trends (*p* > 0.05) observed for all the breast meat quality parameters measured at 24-h post-mortem in response to increasing OMS levels. The SMS0 and CON, breast meat quality parameters were not significantly different (*p* > 0.05).

**Table 8:** Breast meat quality parameters of 15-week-old Boschveld roosters fed with diets containing brown seaweed spent oyster mushroom substrate.

^2^Parameters	^1^Experimntal diets						^3^SEM	Significance		
	CON	SMS0	SMS20	SMS30	SMS40	SMS50		*P*-value	Linear	Quadratic
pH	5.823	5.852	5.881	5.861	5.848	5.865	0.489	0.559	0.602	0.33
Temp (°C)	11.77	12.56	12.31	11.89	11.73	10.50	1.044	0.799	0.294	0.426
L*	65.45	65.69	65.66	67.47	66.37	65.91	0.687	0.343	0.872	0.521
a*	1.472	1.849	1.207	2.085	1.503	1.579	0.287	0.354	0.599	0.689
b*	3.892	2.425	2.916	2.267	2.836	3.116	0.660	0.584	0.658	0.481
Drip loss (%)	12.01	12.45	8.67	12.34	10.81	11.98	1.196	0.231	0.865	0.211
Cooking loss (%)	5.177	6.260	5.584	5.384	5.895	6.088	0.624	0.824	0.917	0.318
Shear force (N)	11.57	11.92	9.797	13.04	13.50	11.00	1.388	0.463	0.889	0.865
WHC (%)	88.66	86.50	88.66	88.25	86.61	85.65	1.683	0.707	0.969	0.066

^1^Experimental diets: CON = a standard grower diet, SMS0 = CON containing 150 g/kg brown seaweed without oyster mushroom spawn inoculation, SMS20 = CON containing 150 g/kg of brown seaweed substrate after inoculating with 20% oyster mushroom spawn, SMS30 = CON containing 150 g/kg of brown seaweed substrate after inoculating with 30% oyster mushroom spawn, SMS40 = CON containing 150 g/kg of brown seaweed substrate after inoculating with 40% oyster mushroom spawn, and SMS50 = CON containing 150 g/kg of brown seaweed substrate after inoculating with 50% oyster mushroom spawn. ^2^Parameters: temp, temperature; L*, lightness; a*, redness; b*, yellowness; WHC, water-holding capacity. ^3^SEM = standard error of the mean.

## Discussion

Brown seaweed can supplement the diets of Boschveld cockerels with nutrients and bioactive substances^27^ required for optimum production. However, higher fibre levels restrict its efficient utilisation. Thus, the inoculation of the seaweed with *Pleurotus ostreatus* fungi can potentially reduce seaweed fibre levels. The absence of dietary effects on mortality indicates that the addition of 150 g/kg brown seaweed as well as the spent mushroom substrate (SMS) does not cause any deaths. The significant week (rooster age) and diet interaction effects on FI, BWG and GF demonstrates that the performance of the birds varied with time (cockerel age), which explains the dietary alterations on FI in weeks 7, 8, 9, 11, 12, 14 and 15. The increase in feed intake shows that the use of *Pleurotus ostreatus* have broken down the lignocellulolytic matrix of the seaweed substrate by producing lignin peroxidase, which oxidizes the non-phenolic lignin substructures through abstracting one electron and generating cation radicals, which are subsequently chemically decomposed^28^. Furthermore, manganese peroxidase produced by the *P*. *ostreatus* oxidizes Mn^+2^ to Mn^+3^, which subsequently oxidizes phenol rings to phenoxyl radicals, resulting in degradation of phenolic and non-phenolic aromatic compounds of the lignin^28^, thereby allowing the birds to consume more feed. This could be seen from the reduced NDF, ADF and ADL levels of the SMS substrates. Feeding SMS-containing diets resulted in a negative linear response for FI in week 13, which was not clear given the increase in FI in the other weeks. Previous research has shown that white-rot fungus (*Phanerochaete chrysoporium*) treatment enhances the CP, EE and ash composition of agricultural by-products by using crude fibre, soluble carbohydrates and their fraction as a carbon source to produce CO_2_ and energy, which is subsequently used together with nitrogen sources for growth and microbial protein synthesis, hence the increase in CP content^29^. This could explain the linear increase in BWG in weeks 8, 11, 14 and 15. The SMS40 treatment promoted the highest BWG than the CON treatment in weeks 6, 7, 9, 11 and 14, which indicated the efficacy of OMS to bolster feed conversion into body mass. This was achieved through the breakdown of fibre by OMS, which also reduced the intestinal viscosity and subsequently allowed for an increase in protein absorption, hence increasing feed efficiency^30^. Further, this could also explain the observed positive quadratic response for BWG in week 5, which showed that the birds fed the CON had numerically lower BWG than the SMS0 and SMS treatment groups. These results corroborate those of Shang et al.^31^, who reported an improvement in average daily weight gain of broilers fed with diets containing 6, 12 and 18 g/kg of *Hericium caput*-*medusae* (Bull.:Fr.) Pers. fungus for 42 days. However, the reason for the linear decline for BWG in week 13 is unknown. The inclusion of the SMS at 40–50% improved GF in weeks 5, 11 and 13, which further confirms its ability to enhance feed efficiency. This could be attributed to the reduced fibre levels of diets further indicating that the birds had access to the beneficial bioactive compounds in the seaweed ^10^. Similarly, Zhang et al.^32^ reported that the supplementation of broiler diets with 3.5 and 7 g/kg of *Aspergillus niger*-fermented *Ginkgo biloba*-leaves improved the feed/gain ratio of the chickens. However, it is not clear why GF showed a linear decline in week 12.

Blood parameters play important roles in the prognosis, diagnosis and treatment of diseases through the assay of various factors influencing haematological and serum biochemical indices^33^. A positive quadratic response was noted for white blood cells (WBC) showing that the SMS-containing diets initially decreased at 20% but increased at 50% OMS level. According to Bhatia et al.^34^, WBC are a diverse group of nucleated cells that play a critical role in immunity and phagocytosis, and therefore in infection defence. The quantity of WBC count normally increases in response to stress or infection, however, a reduction might occur due to bone marrow disease, severe acute disease or other causes^35^. In the current study, CON and SMS50 groups produced higher WBC levels than SMS0 and SMS20 groups, indicating that the latter groups might have endured nutritional problems. Nonetheless, the WBC concentrations for all the treatment groups were within the reported normal range for healthy indigenous chickens^3,36^.

Feeding the SMS-containing diets induced a negative quadratic effect on blood platelets, which participate in immune defence and haemostasis in lower vertebrates such as birds and fish and also play a vital role in reducing blood loss by sealing and repairing damaged blood vessels^37^. Platelets are at the balance of bleeding or clotting events such that excessive bleeding can occur in the case of low platelet levels (thrombocytopenia), whereas thrombosis occurs when platelet levels are high (thrombocytosis)^38^. In the current study, the SMS0 and OMS treatments increased platelet circulation in the blood relative to the CON, which could imply that the SMS0 and SMS-fed birds might have suffered from thrombocytosis. Moreover, the presence of anti-nutritional factors such as lectins, phytic acids, and trypsin and alpha-amylase inhibitors in seaweeds^39^ might have subsequently altered the blood concentration of the roosters. Lymphocytes (L) and heterophils (H) play important roles in adaptive and innate immune defence, respectively^40^. Campbell^41^ reported that healthy birds should have a higher proportion of L than H in their blood, which have an influence on the primary indicator (H/L ratio) of physiological stress^42^. In this study, birds fed the SMS20 had a higher H/L ratio than the CON and SMS50 diets, this is indicative that the SMS20-fed birds might have suffered from minor physiological stress because of the increased H/L ratio when compared to CON-fed birds. On the contrary, Sugiharto et al.^43^ reported dietary influences on mean corpuscular haemoglobin concentration upon supplementing broiler finisher diets with 50, 100 and 150 g/kg of *Chrysonilia crassa* and *Bacillus subtilis* two-stage fermented banana peels.

Feeding the SMS treatments elicited a negative quadratic effect on monocytes, which are the innate immune system phagocytic cells that are responsible for the elimination of pathogens and dead cells^44^. Monocytes play a crucial role in the initiation and cessation of inflammation, primarily through phagocytosis^45^. In the case of bacterial infections or inflammation, monocytes-macrophages are released into the bloodstream to regulate homeostasis. The observed negative quadratic response of monocytes after feeding SMS-containing diets in this study could be explained by the gradual enlargement of the spleen weight, which is regarded as an immediate monocyte reservoir that mobilizes a significant number of monocytes in response to injury^46^. Nonetheless, the SMS-containing diets had similar monocyte values as the CON, which is indicative that the birds did not suffer from any inflammations.

Serum albumin is one of the main sources of serum protein that serves as the most favourable source of amino acids for the synthesis of tissue proteins during the phase of rapid somatic growth of birds, particularly during feed restriction conditions^47^. The observed linear increase in albumin could be due to the presence of phlorotannins in brown seaweed, which might have interfered with protein metabolism because of its binding affinity to proteins^48^. Nonetheless, the similarities among the treatments on ALKP and ALP shows that the birds did not suffer from any liver injuries.

Higher carcass yield has been closely linked to the profitability and viability of poultry production. In this study, slaughter and breast weights improved upon feeding the SMS treatments, which could be attributed to the improvements in growth performance. The SMS treatments had lower NDF, ADF and ADL levels, which could explain the linear decline in caecum weights that is said to enlarge as an adaptive mechanism to handle high fibre levels^9, 49^. This could also explain the observed negative quadratic response in large intestine weight. The spleen is a vital immunological organ in birds, and its size can be utilized as an indicator of immune system responses under different conditions such as infections, inflammation, increasing growth rate and different breeds in males^50^. Tao et al.^51^ reported that birds with enlarged spleen has an infection or disease. The observed quadratic increase in the spleen weights is indicative that the birds might have suffered from an acute condition based on the increased production of platelets (thrombocytosis) and a higher H/L ratio (physiological stress) upon feeding SMS-containing diets. The large intestine weight quadratically decreased with the SMS treatments. This was expected considering that the SMS40 and SMS50 diets tended to have lower fibre content (NDF, ADF and ADL) than the SMS0, SMS20 and SMS30 diets.

The overall consumer approval of meat and meat products is influenced by pH, colour, water retention, texture and sensory qualities^52^. Consumers tend to reject meat items because of unusual appearances that deviate from the expected norm^53^. However, no variations were observed in meat colour across all treatment groups indicating that feeding the SMS-containing diets had no influence on meat appearance. Meat quality indicators such as cooking loss, WHC, drip loss, and tenderness depend on the meat pH value^54^, which is also influenced by glycogen levels in meat muscle before slaughter and the extent to which glycogen is converted to lactic acid after slaughter^55^. Nonetheless, the SMS treatments had no effect on 24-h meat pH (5.82–5.88), which was within the normal meat pH range (5.7–6.1) for poultry meat^56^. This could explain why no dietary effects were observed on the measured meat quality parameters. The shearing force of the meat indicates its tenderness^57^, thus the lack of dietary effects proves that feeding the SMS treatments does not alter meat tenderness.

## Conclusion

Feeding brown seaweed spent oyster mushroom substrate improved growth performance, and slaughter and breast weights, altered the spleen, caecum, large intestine and some of the blood parameters without affecting the general health and meat quality parameters of the roosters. Furthermore, body weight gain was optimised at 20% indicating that the inoculation of brown seaweed with oyster mushroom spawn at this level maximise muscle deposition. The use of oyster mushroom spawn to pre-treat fibrous substrates could be a sustainable approach that can be adopted by the poultry industry.

### Supplementary Information

Supplementary Table S1.

## Data Availability

Correspondence and requests for data supporting this study should be addressed to the corresponding author.
