# Supplementary material for: Dietary incorporation of brown seaweed spent oyster mushroom substrate alters growth performance, physiological responses and meat quality parameters in Boschveld roosters
Source: Sci Rep. 2024 Jun 22;14:14414. doi: 10.1038/s41598-024-65338-0 (PMC11193798; doi:10.1038/s41598-024-65338-0)
Supplement: Supplementary file 1 — Supplementary Table S1. [file 41598_2024_65338_MOESM1_ESM.pdf]

Parameters	Quadratic	R^2	Optimum
[1]WCC ($\times 10^9$/L)	$y = 11.66\ (\pm 0.904) – 0.117\ (\pm 0.078)\ x + 0.004\ (\pm 0.002)\ x^2$	0.483	15%
Heterophils ($\times 10^9$/L)	$y = 7.232\ (\pm 0.690) + 0.122\ (\pm 0.060)\ x – 0.003\ (\pm 0.001)\ x^2$	0.191	20%
Platelets ($\times 10^9$/L)	$y = 28.85\ (\pm 2.109) + 0.300\ (\pm 2.183)\ x – 0.010\ (\pm 0.004)\ x^2$	0.443	15%
Lymphocytes (%)	$y = 21.50\ (\pm 4.77) – 1.007\ (\pm 0.413)\ x + 0.031\ (\pm 0.008)\ x^2$	0.541	16%
Monocytes (%)	$y = 11.11\ (\pm 1.156) + 0.215\ (\pm 0.100)\ x – 0.006\ (\pm 0.002)\ x^2$	0.316	18%

Supplementary Table S1. Regression equations for haematological parameters of 15-week-old Boschveld roosters fed with diets containing brown seaweed spent oyster mushroom substrate. WBC = white blood cell.
